# The Principles and Practice of Surgery

**Published:** 1852-10

**Authors:** 


*The Principles and Practice of Surgery. Illustrated by three hundred and sixteen engravings on wood.* By WILLIAM PIRRIE, F. R. S. E., Regius Professor of Surgery in the Marischal College and University of Aberdeen, Surgeon to the Royal Infirmary, &c. &c. Edited with Additions by *John Neill,* M. D., Surgeon to the Pennsylvania Hospital, Demonstrator of Anatomy in the University of Pennsylvania, &c. Philadelphia: Blanchard & Lea, 1852. 8vo. pp. 784, including Index.

The first of the two works above presented has been some time awaiting notice on our table. At any other than the dog-day season, it would certainly have met with the immediate attention which the offspring of such high parentage demands. As it was, however, the labor of delving through seventy-five voluminous and desultory lectures, comprising nearly eight hundred pages of surgical wisdom and experience, was more than our energies were equal to until the recent arrival of a younger and less formidable applicant, in the second work announced, has roused us to the task.

Mr. Cooper's "Lectures" cannot be new to many of our readers, since they were originally published in the London Lancet shortly after their delivery to the lecturer's hospital pupils, and thus circulated widely in this country. They require, therefore, a less particular examination at this time than we might otherwise feel bound to give them. Our author informs us that he has spared no pains in the preparation of his work, and that a considerable addition has been made to it in its present form. His object was "to furnish a useful compendium of surgery, in which the student may meet with a clear account of the practice of that science, established not only on my own experience, but likewise upon the best acknowledged authorities." He previously tells us that "it is not without mature consideration that I have determined upon publishing the present volume; but I have come to the conclusion that as its contents are of a practical character, embodying the experience of twenty-five years, during which I have occupied the position of surgeon to Guy's Hospital, it would be found useful not only to the student, but also to those who have entered upon the practice of their profession." Notwithstanding these claims, however, he wishes it to be "borne in mind that it has not been my intention to write a systematic work on the elements of the science of surgery."

Nevertheless, with every due allowance, and putting "the elements of the science" and "the abstract principles of the science," as he phrases it in another place, altogether out of the question, it must be said that his "practical book" makes but a very sorry figure as the result of a quarter of a century's opportunities as a surgeon and clinical teacher in the wards of Guy's Hospital, to say nothing of the many other invaluable advantages of the author as a military surgeon in at least one Peninsular campaign, and as the nephew and associate and successor in practice and teaching of Sir Astley Cooper. We do not pretend to say that there is not a great deal of interesting and instructive reading in these lectures—much that may be noted and acted on with profit by the practitioner and teacher, even more than by the student—much, too, that may not be found so strikingly or usefully exhibited in other works; still the apprehension with which recent experience has inclined us to regard these "practical books," is not lessened by the advent of Mr. Bransby Cooper's publication. Supposing it to comprehend to a sufficient extent "the general principles of science upon which the practice is based," as he proposes, and admitting it to be the useful compendium of surgery intended, it is yet very far from being the clear and comprehensive account of the practice itself which we have a right to look for from such a source, or which the student ought alone to have. The rambling, diffuse and contradictory style of many portions of these lectures, and the strangely loose and inaccurate assertions frequently occurring in them, have become so well known and so generally commented on, that we feel less obliged to quote examples in this place. We may refer, however, to the lectures (6 and 7) on Hydrophobia and Tetanus. He says, "Of all the diseases to which the human system is liable, there seems to me none so appalling as that produced by the bite of a mad dog." Now, in the very next lecture he contradicts himself, and makes a most extravagant assertion, in the following language: "The disease which forms the subject of this chapter is as appalling as that which occupied our attention in the last lecture, and unfortunately as incurable(!)—I allude to Tetanus." Hydrophobia is stated in his previous lecture to be beyond "any rational hope of cure," (p. 80;) and although he professes to consider tetanus no less incurable, yet in the course of his lecture on the subject, he tells us that he had an opportunity of witnessing symptoms of tetanus resulting from the injection of a hydrocele, in which recovery took place under the use of large doses of calomel and opium, with turpentine enemata, (p. 84.) On the next page we read that "cases are recorded which have been successfully treated by the complete division of a wounded nerve, and by the removal of splinters of wood;" while a little further on, in remarking on a case which he reports in full, he says: "Notwithstanding the fatal result of this case, there seems just reason to consider that the tetanic symptoms were at once put a stop to by the removal of the source of irritation; it is clear that the patient fell a victim to phlebitis." Again, in lecture 46, on Amputations (p. 683,) he observes: "Baron Larrey, during the war in Egypt, amputated in several cases after symptoms of tetanus had commenced, and this practice was attended with sufficient success to induce him to recommend it." Having thus established—meagerly, indeed, and very oddly, for a man of his authority—the fact which no well-informed surgeon would deliberately deny, the curability of tetanus, he, in the face of his own implied precept, concludes his chapter, as he began it, to the following effect: "Surgery, I fear, has done as little for the cure of this disease as physic, although there are recorded cases of persons having recovered when the amputation of limbs has been performed after tetanic symptoms had commenced, and where they had yielded upon the division of wounded nerves; but still, these operations have so frequently failed, that had a true statistical account been kept, I suspect but little more could be said for surgical than for medical treatment, when once the disease is developed in an acute form. So little light has as yet been thrown upon the true nature of the disease, that the medical practitioner is without the least guide to direct him in his practice. Tetanus, like hydrophobia, has hitherto defied the efforts of the physician," &c. &c.

A glance over the lecture on gun-shot wounds, which follows that on tetanus, affords us another sample of our author's style of teaching. The following will give some idea of his mode of jumping to a conclusion. "Any violent concentrated force may produce the same results as a gun-shot wound, and therefore all extreme contusions from machinery and railroad accidents offer precisely similar pathological considerations. Monographs on gun-shot wounds, or, indeed, on any subject, are therefore somewhat dangerous, as they lead pupils, more especially, to consider the subject as separated from the general principles of surgery." Now we will not stop to quarrel with his vague and sweeping mode of expressing the analogy between injuries arising from fire-arms and "all extreme contusions from machinery and rail-road accidents," but we must beg leave to demur entirely to the plea in condemnation of monographs, as "dangerous," because forsooth the thoughtless student may misunderstand their objects and the meaning of their titles.

This chapter on gun-shot wounds contains among other contradictions, one which, although unimportant in itself and doubtless an inadvertence, is so very marked as to have attracted general attention. Whether an accident or not, it is worth noting as a portion of the evidence against the general accuracy of the book, and of its consequent unfitness for the student to whom it is especially addressed. A volume of lectures emanating from such an influential source and supposed to be revised with the greatest care, after having been first rehearsed in the lecture room and subsequently reported for the press and published in a medical periodical, must be expected to be especially reliable and free from errors, small as well as great, or to be held correspondingly accountable. We require from the veteran what we could not hope for from the beginner; and no one should complain if the short-comings of the former be visited with the utmost rigor of the law.

To return to our subject proper, on page 96 it is stated that "the wound made by the entrance of the ball is small, and its lips are inverted, discolored and valvular, while the opening through which the ball has made its escape is much larger, with an everted and ragged edge." This is the old-fashioned account, and a true enough one too in many cases. Now for the later story, which is here announced without the slightest allusion to the dictum previously given. "Many different statements have been made respecting the appearance and comparative size of the openings made at the entrance and exit of the bullet. It has been said that the hole by which it enters is smaller and cleaner than that by which it leaves the body, which is ragged and more gaping. This does not, however, seem to be correct; the opening by which the ball enters appears to be generally somewhat the larger of the two. But, in fact, there is so little difference between them that unless the direction of the shot were previously known, it would be impossible to say by which opening the ball entered, or by which it left the body."

It is to be presumed that those who swear by Mr. Bransby Cooper must split this "little difference" for themselves, until further orders—they might as well toss up for it like school boys, so far as his directions are concerned. And thus it is to a greater or less extent throughout the volume. The valuable matter, of which of course there is in various shapes a really large amount, might be compressed with advantage to the whole book into two-thirds if not one half of the present unmanageable bulk of the latter; while in spite of the author's second thought, adverse to "the assistance of wood-cuts," we think a few effective diagrams and other illustrations properly distributed would have increased the usefulness quite as much as the attractiveness of a crowded and tedious mass of letter press that has nothing now to break in upon its monotony or lighten up its dulness. We do not consider ourselves bound to pursue this examination further, and will take leave of Mr. Cooper with a quotation of which no one need complain. We have taken it at random as an illustration of one of the good points in the book—the caution of the careful and experienced surgeon. Obviously wise and proper as the precept here presented is, it is yet too often shamefully neglected, not only in actual practice but in the books themselves.

"After what has been said as to the tendency to erysipelas following wounds of the scalp and skin of the face, let me urge the necessity of caution in undertaking even trivial operations on these regions of the body, without first having duly prepared the patient for the effects they invariably produce in the system. In some cases the surgeon may be requested to remove small encysted tumors from the scalp—an operation so trivial that it may be executed by a mere tyro in the profession; but even the most experienced and skilful surgeon may risk the life of a patient, and his own reputation, by want of a little precaution."

"Never, I say, should such a task be undertaken without the actual state of the patient's health being first well ascertained, as to the absence of any organic disease, the condition of the bowels, state of the urine, and natural performance of the functions essential to a healthy state of the body." After detailing three different cases in illustration of the importance of the principle, he goes on to say, "such cases as these show the necessity for doing everything which the science of surgery can command to place our patient in the greatest state of security, before we subject him to any surgical operation, and even then never to promise that any operation, however simple, will be perfectly free from danger; for depend upon it, it is as unwise to treat slightingly the most trifling incisions of the skin, as it is dishonest to attach to an operation more importance than it fully deserves."

Mr. Pirrie's work is one of much more moderate pretensions than Mr. Cooper's, and yet is, in our view, not only a vastly superior book of its kind, but one that is better altogether, and likely to be much more useful as well as more popular.

It is not offered as a "compendium of surgery," but simply as a compendium of its author's lectures, published in compliance with the long expressed wish of his pupils to be furnished with such a guide. It is not especially addressed to any class of readers, or placed in competition with any of its kind. And yet with a very little extension and other alteration it might be so admirably fitted for the purposes of a text-book, as to outstrip in popularity any competition that could be brought against it at the present time.

His endeavor in the preparation of his volume was "to combine simplicity of arrangement and conciseness and clearness of description with the elucidation of sound principles and practice." An examination of the different chapters will convince any one that, as far as he has gone, he has been remarkably successful in the execution of his task. We say, as far as he has gone; for unfortunately several important topics are not treated of at all, while perhaps as many more are hurried over or barely mentioned. Some of the articles, at the same time, are occupied with subjects which are evidently such favorites with the author as to have received an undue share of his attention at the expense of others. In this way some portions of his book have been so thoroughly worked up, and in themselves exhibit such excellent specimens of effective condensation, whilst they convey the very latest information in connection with their subjects, that we decidedly regret that our author's modesty has so limited his plan. It is only necessary for him to develope his discussion of many topics to a more proportionate length and fulness, and to introduce others which have not yet found a place, in order to make his book the very best of its kind in the English language.

The defects of Mr. Pirrie's volume affect rather its completeness and uniformity as a general work on Surgery than its value to the student as a most companionable guide book in his study of the topics to which the several chapters are devoted.

These chapters are twenty-five in number, each being distinct in itself, and occupied with some particular disease or injury, or class of diseases. They may be briefly enumerated with their titles in the following order: 1, Inflammation and its Results, a comparatively meager chapter, especially in its account of the treatment and of the Results, and yet well brought up and furnished with the most recent views. 2, Erythema and Erysipelas, also a meagre chapter. 3, Wounds, rather more full, but still not as full as we think it ought to be—except in the section on gun-wounds, which is excellent. 4, Burns, in all respects a capital chapter. We would be glad, were there room here, to quote a portion of this article, by which we might afford some idea of the author's best style, both of matter and language. This chapter is extended to twenty-one pages, while that on wounds is compressed into thirty-one; and chapter fifth, on fractures, has but seventy-five. The last named chapter is deficient, not only in its discussion of certain fractures, but in its omission of others altogether. Dislocations come next in order, and take their place a little more at length than fractures. Succeeding them we have affections of the Osseous system, a capital chapter; Diseases of the Joints, also admirable; Curvatures of the Spine, an instructive and beautifully illustrated chapter; Talipes, also good; Affections of the Arteries and Brain, one of the very best chapters; Hernia, not inferior in any respect to the last; Wounds of the Abdomen; Calculous disorders, a full and attractive chapter; Affections of the Testicle, rather defective; Affections of the Genito-urinary Organs, still more incomplete; Amputations and Resections, very full in most respects, but no mention of the circular method of amputation; Deligation of Arteries, full and richly illustrated; Affections of the Rectum, rather incomplete; Affections of the Eye and its appendages, a very good summary; Affections of the Nose, not very full; Affections of the Mouth, Throat and Windpipe, not more satisfactory; Tumors, full, well arranged and well illustrated; and lastly, a rather brief chapter on Affections of the Breast. With this hasty comparative review of its contents, and with a solitary extract, we regret to be obliged to leave Mr. Pirrie's book. We would be very glad to be at liberty to discuss many matters of interest in different portions of the volume, and to dwell especially on several of the chapters which we are inclined to regard as models in their way. We must content ourselves, however, with the following quotation, which, although taken pretty much at random, will at least serve to give some idea of our author's style and powers of lucid condensation:

" *Treatment* [*of Acute Abscess.*]—To remove general and local causes of excitement and irritation, and to promote the approach of the matter toward the surface, are important indications in the treatment of abscess. The best means of fulfilling these indications, are the strict observance of antiphlogistic regimen, perfect rest of the affected part, strict maintenance of a proper attitude, the removal of all sources of irritation, as well as of tension, or pressure, and the diligent use of warm emollient poultices, hot fomentations being applied each time the poultice is removed. Purulent matter having once been formed, it may be stated as a general rule in the treatment of acute abscess, that the grand indication then to be fulfilled is the early and free discharge of the matter. In some circumstances very early attention to this rule is of the utmost importance; as in abscesses under dense aponeuroses, and under thick fasciæ, (as, for example, under the temporal aponeurosis or under the tendon of the occipito-frontalis muscle, or under the fascia of the thigh, of the leg, of the arm, or of the fore-arm, or under the palmar or plantar fasciæ), within tendinous sheaths, as in paronychia tendinosa, or underneath the periosteum, as in paronychia periostei, or under the pericranium, in the proximity of bones, in the natural cavities, or in the texture of bones; also in abscesses arising from the extravasation of irritating fluids, as collections of matter caused by the extravasation of urine into the cellular tissue of the perineum and scrotum; abscesses in parts abounding with cellular tissue, when there is great risk of the spreading of the inflammation, or in situations where there is danger of making their way into some of the natural cavities, as into the chest or abdomen, or the joints; or such as are likely to occasion injury by pressing upon or impending the functions or important parts, as the trachea, the pharynx, the urethra; or abscesses, in highly vascular and sensitive parts, where the pain of an abscess is often most excruciating. With scarcely more than one exception, early and free opening of an abscess is the proper course; but in the above-mentioned conditions, it is peculiarly necessary to adopt this proceeding at the earliest possible period after we are certain of the actual existence of matter; for not only are time and suffering saved and tissue preserved by its adoption, but by its neglect the danger of most destructive local results is increased, and, in some circumstances, even the loss of life itself may result. Almost the only condition in which it is proper to delay opening, is in cases of glandular enlargement, in which, when other means have failed to produce absorption, and suppuration has occurred, the opening should be deferred, that the pressure of the matter may more effectually secure the disintegration and breaking up of the glandular structure, and thereby favor its removal.

" Of the various methods of opening abscesses I shall refer to only two; namely, by means of a bistoury, and by means of caustic. The former is preferable, except in two conditions, presently to be mentioned. It consists in making a *free, direct* opening in a depending situation, and as already stated, at an early period. In the event of the matter making its way in a different direction, a second opening should be made, which, from often being opposite to the first, is called a counter-opening. By making an early, large, direct opening in a depending situation, and keeping it open while matter continues to be secreted, the formation of sinuses, loss of substances, and disintegration are generally prevented, and the desired result is obtained more speedily, and with less suffering than it would be by any other proceeding.

"The two conditions in which opening by means of caustic is preferable, are the following:

"1. In small abscesses, partaking of a chronic character, where the integuments are attenuated in consequence of the opening having been delayed, or where they are in a diseased state. In such cases, the integuments are too much weakened to take on the necessary action for uniting with the subjacent parts; and as no healing process takes place until they are destroyed by ulceration, it is better to destroy them at once, and make a free opening by means of caustic. For this purpose the potassa fusa is preferred, and is applied so as to destroy the whole of the diseased and thinned integument.

"2. In cases of glandular enlargement in the state of abscess, in which condition the caustic should be used very freely, and be pressed into the gland in different situations, so as to lead to the action by which the diseased structure may be separated and removed." pp. 48, 49.

The additions of the American editor are to the purpose generally, and cannot fail to increase the value of the work, especially to readers in this country. The publishers have done their duty handsomely in the general getting up; and in regard to paper, typography and wood-cuts, have fallen little, if any, short of the original, whilst in the increased amount of text and illustrations, they have really gone beyond it. We cordially recommend their publication as already one of the best text-books on Surgery yet published in the country, and likely to become, in the new edition which must soon be called for, equal if not superior to any now extant.

